# Oral Focal Mucinosis of the Tongue: Case Report and Review of the Literature

**DOI:** 10.7759/cureus.67882

**Published:** 2024-08-26

**Authors:** Ryota Kobayashi, Hideaki Hirai, Satoshi Maruyama, Jun-ichi Tanuma, Kei Tomihara

**Affiliations:** 1 Division of Oral and Maxillofacial Surgery, Faculty of Dentistry & Graduate School of Medical and Dental Sciences, Niigata University, Niigata, JPN; 2 Oral Pathology Section, Department of Surgical Pathology, Niigata University Hospital, Niigata, JPN; 3 Division of Oral Pathology, Faculty of Dentistry & Graduate School of Medical and Dental Sciences, Niigata University, Niigata, JPN

**Keywords:** pathological diagnosis, surgical excision, oral cavity, tongue, oral focal mucinosis

## Abstract

Oral focal mucinosis (OFM) is an oral mucosal lesion characterized by focal mucosal accumulation that rarely occurs on the tongue. This report describes a rare case of OFM on the right side of the tongue in a 71-year-old female patient. The clinical features of OFM have not been well defined, making it difficult to differentiate it from other lesions based solely on clinical manifestations; therefore, histopathological examinations are necessary. Although OFM on the tongue is rare and has a good prognosis with resection, it should be considered as differential for painless mass lesions in the oral cavity.

## Introduction

Oral focal mucinosis (OFM) is an oral mucosal lesion characterized by asymptomatic focal mucous accumulation and presents with clinical manifestations such as a benign tumor.

It was first reported by Tomichi et al. in 1974, and is considered a lesion similar to cutaneous focal mucinosis of the oral mucosa [1]. Although the etiology remains unknown, it is thought to be caused by overproduction of hyaluronic acid by fibroblasts [2]. Clinically, OFM usually presents as a painless, sessile, firm nodule that is the same color as the surrounding mucosa [2,3]. Due to a lack of distinctive clinical features and its rarity, it is often misdiagnosed as other mucosal diseases. Histopathologically, OFM is characterized by well-localized myxomatous tissue without a capsule, surrounded by denser, normal collagenous connective tissue. It typically occurs in adults in their fourth to fifth decades of life, and is more common in women than in men. The gingiva and hard palate are frequently affected sites, and the tongue is rarely affected. To the best of our knowledge, only nine cases of OFM of the tongue, including ours, have been reported to date. This paper reports the case of a 71-year-old female patient with OFM of the tongue and reviews the relevant literature.

## Case presentation

**Figure 1:**
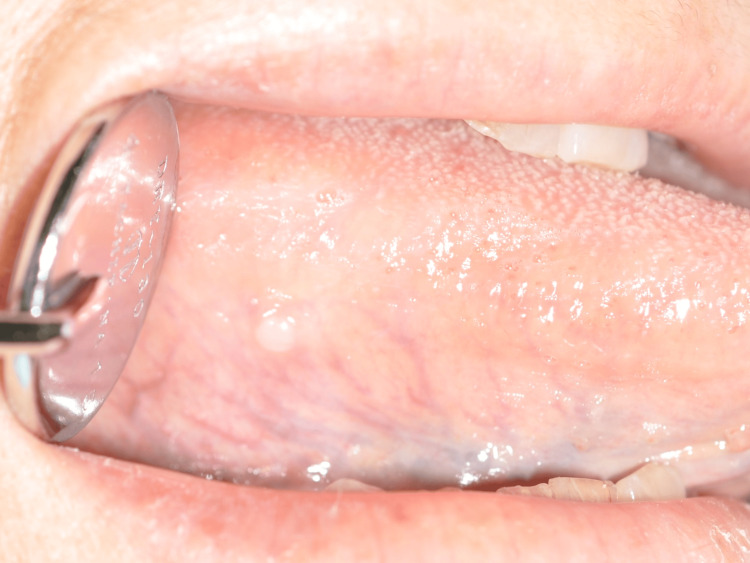
A 2×2 mm, hard, non-mobile, painless, hemispherical mass with a smooth surface is observed on the right tongue border.

A 71-year-old Japanese woman was diagnosed with painless eruptions on the right side of her tongue in January 2018 and was referred to our hospital in July 2018. Intraoral findings revealed a hemispherical mass on the right side of the tongue with a 2 × 2 mm smooth, white mucosal surface (Figure 1). The mass was hard and non-mobile, and no contact pain or tenderness was noted. The patient's medical history included hypertension, dyslipidemia, and gastric polypectomy. She had no habits that irritate the tongue, such as grinding or chewing, and had no history of smoking or alcohol consumption. The clinical diagnosis was a fibrous polyp of the tongue, and treatment was planned as an excisional biopsy without imaging studies because of the tiny lesion.

In August 2018, a tumor excision was performed under local anesthesia with a 3 mm safety margin and primary closure. The patient was followed up for five years, with no evidence of disease recurrence.

**Figure 2:**
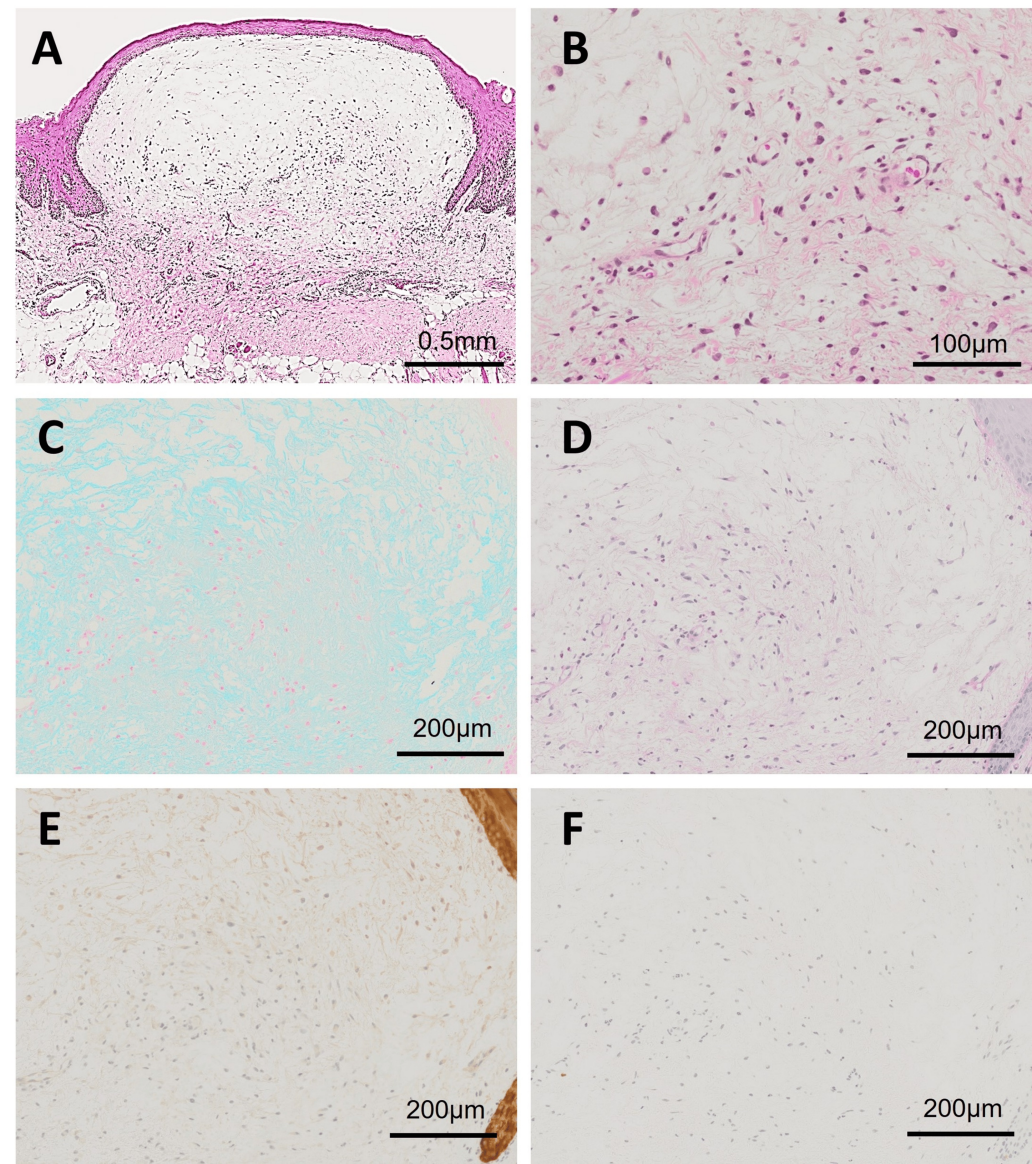
(A) Low-power view shows a well-localized mucous-like tissue deposition with a clear border with the surrounding area and no capsular structure. (HE) (B) High-power view shows abundant stellate cells corresponding to fibroblasts in the mucus matrix. (HE) (C) The mucus matrix just below the mucosal epithelium is positive for Alcian blue staining. (D) The mucus matrix just below the mucosal epithelium is non-positive for PAS. (E) No pan-keratin (AE1/AE3)-positive epithelial cells are observed in the mucosal matrix. (F) No S-100 protein-positive neuronal cells are observed within the mucous matrix. HE: Hematoxylin and eosin, PAS: Periodic acid–Schiff

Histopathologically, the lesion was a mass extending outward from the surface of the tongue mucosa, with mucous-like material deposits mainly in the lamina propria and submucosal layers, which were well demarcated from the surrounding tissue but without capsular structure (Figure 2A). On the deep side of the mucous-tissue with material, penetration of the mucous-like tissue into the surrounding collagen fiber bundles was observed; no salivary gland tissue was observed in the surrounding area (Figure 2B). The subepithelial mucous-like tissue was positive for Alcian blue (Figure 2C) and negative for Periodic acid-Schiff (PAS) (Figure 2D), AE1/AE3 (Figure 2E), and S-100 protein (Figure 2F) in spindle-shaped fibroblast-like cells within the mucous matrix. Based on these findings, OFM was diagnosed.

## Discussion

OFM is a rare oral mucosal lesion characterized by focal mucous accumulation, most commonly occurring in the gingiva. Including this case, a total of 108 cases have been reported in previous English literature, with a male-to-female ratio of 1:1.8, indicating a higher prevalence in females. The age range of affected individuals spans from one to 78 years (mean, 42 years), with occurrences being uncommon in children and adolescents [2].

The most affected site was the gingiva in 70 cases (64%), followed by the palate in 17 cases (15%), tongue in nine cases (8.3%), buccal mucosa in five cases (4.6%), oral floor in three cases (2.7%), lip in one case (0.9%), and others in three cases (2.7%), consistent with reports indicating that the jaw bone-attached mucosa is more susceptible [1,2]. There have been nine cases of OFMs in the tongue, including the present case, with ages ranging from 45 to 71 years (mean, 60 years), a male-to-female ratio of 2:1, and a mean length of 5 mm (Table 1) [1,3,4,7-10]. In contrast, the 99 patients with OFM occurring at sites other than the tongue had ages ranging from one to 77 years (mean, 42 years), a male-to-female ratio of 1:2, and a mean length of 13 mm. Therefore, OFM of the tongue tends to be more common in older patients, particularly males, and smaller in size compared to lesions occurring at other sites.

**Table 1:** Reported cases of oral focal mucinosis of the tongue

Case	Authors	Age (years)	Sex	Ethnicity	Site	Size (mm)	Disease duration	Clinical Symptom	Clinical presentation	Color	Clinical diagnosis	Treatment	Recurrence (follow-up period)
1	Tomichi et al. (1974) [1]	45	M	white	tip of tongue	-	2M	-	-	-	mucocele	-	-
2	Buchner et al. (1990) [8]	50	M	-	ventral tongue	4×4	2M	-	-	-	-	excision	no(not stated)
3	Soda et al. (1998) [9]	68	M	-	anterior ventral tongue	-	3Y	no	Soft swelling	normochromic	-	excision	no(not stated)
4	Aldred et al. (2003) [7]	55	M	-	tip of tongue	5×5	3M	-	Hard, pedunculated	grey/white	fibroepithelial polyp	excision	no(8M)
5	Pacifici et al. (2012) [10]	62	F	-	tip of tongue	5×5	-	no	Round-shaped, hard, non-mobile	pink	-	excision	no(not stated)
6	Higuchi et al. (2016) [4]	67	M	Asian	tip of tongue	3×3	1M	no	Hemispherical mass, hard, non-mobile	white	fibroma	excision	no(3Y3M)
7	Silva et al. (2020) [3]	62	M	Non-white	tongue	3×3	-	-	Hemispherical mass, smooth, fibroelastic	red	fibrous hyperplasia	excision	no(not stated)
8	Silva et al. (2020) [3]	63	F	-	tongue	10×10	48M	no	Hemispherical mass, smooth, fibroelastic	normochromic	fibrous hyperplasia	excision	no(not stated)
9	Present case	71	F	Asian	side of tongue	2×2	6M	no	Hemispherical mass, fhard, non-mobile	normochromic	fibroma	excision	no(5Y6M)

The etiology of OFM is thought to be mucin accumulation caused by overproduction of hyaluronic acid by fibroblasts, although evidence is still lacking [2,3]. Previous reports have described its occurrence at the site of contact with the acute margin of the tooth and in the gingiva of the maxillary central incisor in patients who underwent rapid maxillary expansion [4], suggesting that traumatic or mechanical tissue irritation may have caused hyaluronic acid hyperactivity in the fibroblasts [3]. However, there was no obvious mechanical irritation, such as sharp edges of the teeth, in our case.

Because of the variety of clinical manifestations, lack of specific clinical features, and rarity of the lesions, there have been no previous reports of OFM based on clinical findings. Silva et al. reported that the clinical features of OFM are not always consistent with the characteristics of an asymptomatic mass lesion coated with a normal mucosal surface and normal color [3]. In previous reports, multiple clinical findings have been demonstrated, including bluish-purple, irregular, granular, and ulcerated lesions. Therefore, the initial diagnosis varies widely, including fibromas, papillomas, pleomorphic adenomas, pyogenic granuloma, lipoma, epulis, giant cell granuloma, and solitary fibrous tumor (SFT) [2,3]. The patient was clinically diagnosed with a fibrous polyp.

Histopathologically, OFM is characterized by submucosal mucin accumulation with a well-defined border and no capsule and is reported result from excessive hyaluronic acid production by fibroblasts instead of collagen fibers [2-4]. Thus, it is important to distinguish OFM from diseases with a myxomatous substrate, such as odontogenic myxomas, peripheral nervous system tumors such as nerve sheath myxomas, and SFT. The mucus matrix of OFM is acidic mucin, which is negative for PAS staining and positive for Alcian blue, whereas that of odontogenic myxomas is negative for Alcian blue [5]. AE1/AE3 is useful for differentiating epithelial tumors, and the S-100 protein is useful for differentiating peripheral nervous system tumors. SFTs, classified as an intermediate grade by the World Health Organization, are most commonly found in the buccal mucosa and tongue of the oral cavity. Their clinical presentations are similar; therefore, careful differentiation is required [6].

Surgical excision was performed to treat OFM in all cases described in the literature (Table 1) [1,3,4,7-10].

This disease has a low risk of recurrence and a good prognosis after complete excision, with only one case of recurrence reported on the upper lip [3]. Owing to the rarity and limited information on this disease, there may be cases that were coincidentally resected without an exact diagnosis.

## Conclusions

OFM is a rare, benign oral lesion characterized by mucous accumulation, often found in the gingiva but extremely rarely in the tongue. Diagnosing OFM involves histopathological analysis to differentiate it from other similar lesions. Surgical excision is the common treatment, yielding good prognosis with minimal recurrence risk. This report highlights the need for documenting and evaluating future cases of OFM in the tongue to refine treatment strategies and improve outcomes.
